# Pediatric fibroma in maxillary sinus following nasal trauma: A case report

**DOI:** 10.1016/j.ijscr.2019.05.062

**Published:** 2019-06-11

**Authors:** M. Akdağ

**Affiliations:** Dicle University Medical School, Department of Otolaryngology, Diyarbakir, Turkey

**Keywords:** Fibroma, Maxillofacial trauma, Surgery of maxiller fibroma

## Abstract

**Introduction:**

Fibroma is a benign tumor of the connective tissue and is classified among the fibrous and fibrous histiocytic lesions. In the pediatric age group, both the differential diagnosis and surgery of maxillary swellings are difficult.

**Case reports:**

A 26-month old girl applied with swelling and malformation in the nose. She complained of swelling in the right cheek, nasal obstruction and dyspnea during the night time. Her family stated that she had a head trauma 45 days before the application. We observed a rigid mass in the lateral side of the right nasal region consistent with severe edema. The pediatric endoscopic examination displayed a passage obstruction and an expansion of the lateral wall towards the septum. We performed right Caldwell-Luc approach with the endoscopic transnasal approach. The pathological examination with the light microscope displayed dense and thick collagen fibers and fusiform fibroblasts mixed up with these collagen fibers.

**Discussion:**

Fibroma and fibroma like lesions are rare and difficult to diagnose. In the Pubmed database, we did not encounter any large-scale study focused on this type of tumors except for some case reports of fibromyxoma. In respect of the findings in the literature, along with the pathological criteria, the clinicopathological correlation and radiological imaging may facilitate the diagnosis. Furthermore, a good cooperation with the patient and his/her family and a reliable history like trauma are also critical.

**Conclusion:**

The possibility of trauma should be definitively questioned during the history of pediatric patients, who applied with a maxillofacial swelling with the presence of persistent edema and differential diagnosis for the soft tissue tumors like fibroma should be considered.

## Highlights

• Fibroma and fibroma like lesions are rare and difficult to diagnose in pediatric ageas.
• In the Pubmed database, we did not encounter any large-scale study focused on this type of tumors except for some case reports of fibromyxoma.
• Management of fibroma in the maxillofacial area is surgical excision.
• If there is a persistent maxillofacial swelling and edema with history of trauma in the pediatric age you should be considered differential diagnosis for the soft tissue tumors like fibroma.

## 1 Introduction

Benign fibro-osseous lesions represent a group of lesions that emerge usually on chin and facial bones as a result of replacement of the bone tissue with the fibrous connective tissue and bone and cement in different amounts. Although the histological characteristics may be useful for the diagnosis of some lesions, additional detailed clinical and radiographic examinations are usually required for a definitive diagnosis [[1], [2], [3], [4], [5], [6]]. As a fibro-osseous lesion, fibroma consists of fibroblasts spread throughout the dense collagen tissue. In spite of the low mitosis rate, an infiltrative proliferation is usually the rule. Fibroma does not exhibit atypia. As it may emerge after traumas (spindle-cell tumor, sarcomatoid degeneration, cemento-ossified fibroma, desmoid tumor etc.), differential diagnosis is relatively difficult. Certain disorders such as benign and malign fibrous lesions, angiofibroma, benign and malign smooth muscle cell tumors, and neurogenic tumors should be also considered. Surgical intervention is the standard treatment. This study was stated according of SCARE criteria [7].

## 2 Presentation of case

A 26-month-old girl presented to our outpatient clinic with the complaint of swelling and malformation on the nose. She complained of swelling in the right cheek, nasal obstruction, and nocturnal dyspnea. Her family explained that she had a head trauma approximately 45 days before presentation. Her family had immediately applied ice on the trauma area and fetched her to an ENT specialist. According to the anamnesis, the ENT specialist prescribed a medical treatment after the clinical examination. Thereafter, a plastic surgeon performed an incision on the lesion to drain blood depending on a prediagnosis of hematoma; however, the swelling did not regress.

**Fig. 1:**
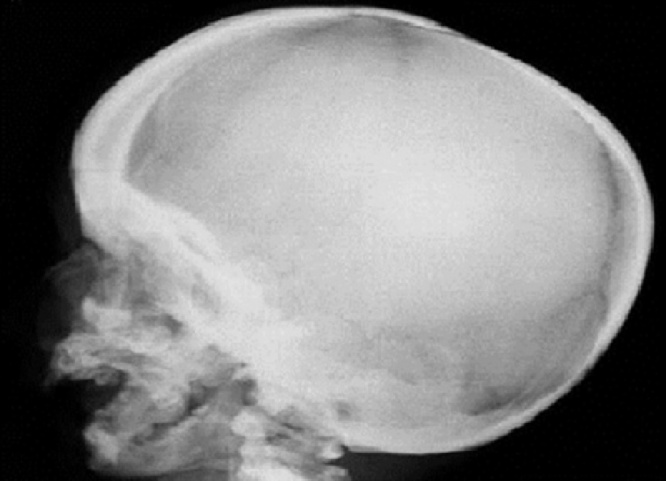
The direct x-ray examination did not show a prominent fracture in the nasal region.

**Fig. 2:**
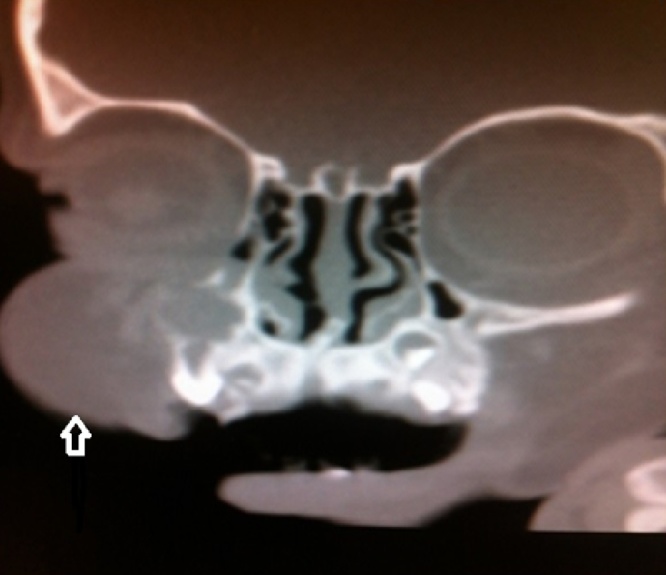
The computerized tomography of the paranasal region displayed a cystic mass, which was expanding to the right lateral bone towards the nasal passage and extending towards the infraorbital region in the superior segment and towards the dental root in the inferior segment and 10 × 8 mm diameter as measured.

**Fig. 3:**
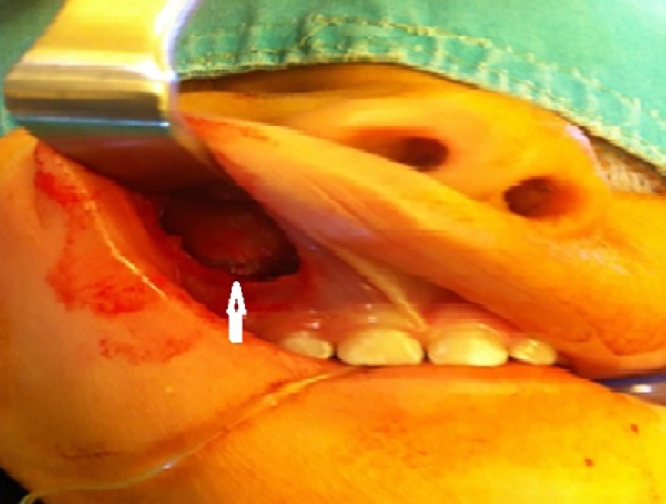
Transnasal endoscopic view of gingivobuccal incision as right Caldwell-Luc procedure.

**Fig. 4:**
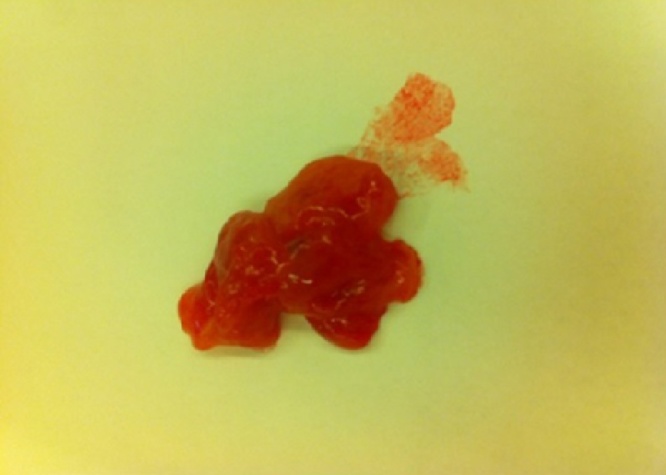
The view of extracted of mass. The size of the lesions near was 10 × 8 × 6 cm as diameter.

**Fig. 5:**
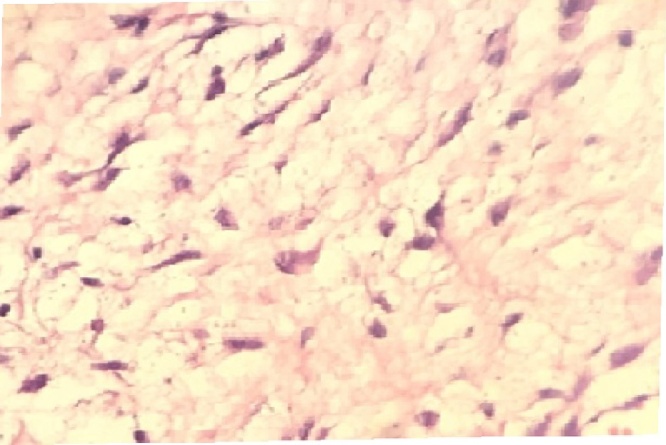
The histological sections with light microscope image from mass that was displayed dense and thick collagen fibers and fusiform fibroblasts mixed up with the collagen fibers which compatible fibroma (H–E staining, 200× magnification).

At the time of presentation to hospital, we observed a rigid mass on the lateral side of the right nasal region consistent with edema. The skin color was slightly changed and we noticed an incision scar on the lesion. The rhinoscopic examination showed a narrowed passage on the right side. As the obstruction observed during the endoscopic examination did not regress with the decongestant impregnated embedded gauze pad, we suspected that it would be a turbinate hypertrophy. We observed that the lateral wall expanded towards the septum. The direct x-ray examination did not show a prominent fracture in the nasal region (Fig. 1). The CT examination of the paranasal region displayed a cystic mass, which was expanding to the right lateral bone towards the nasal passage and extending towards the infraorbital region in the superior segment and towards the dental root in the inferior segment and 10 × 8 mm diameter as measured (Fig. 2). We planned a diagnostic and therapeutic surgical intervention after we had obtained the informed consent from her family. We were performed external and endoscopic surgery as combined approached. During the intervention carried out with a 0-degree endoscope, we noticed that the maxillary sinus was filled with soft tissue. Transnasal antrostomy and sinus aspiration did not deliver a considerable amount of fluid. Therefore, we extended the endoscopic transnasal intervention with gingivobuccal incision as right Caldwell-Luc procedure Fig. 3). Thus, we were able to access the lesion. We were observed the pink colored, fragile and partially hemorrhagic lesion in the maxillary sinus and extending to the infraorbital eye muscles. The mass was extracted that while the eye muscles were preserved. The size of the lesions 10 × 8 × 6 cm as diameter (Fig. 4). The pathological examination with the light microscope displayed dense and thick collagen fibers and fusiform fibroblasts mixed up with the collagen fibers Fig. 5. Taking the absence of atypia in the fibroblasts into consideration, the patient was diagnosed with fibroma.

## 3 Discussion

Fibroma is a benign tumor of the connective tissue and is classified among the fibrous and fibrous histiocytic lesions. Pure benign neoplasms of the fibrocyte are rare in any part of body. A fibroma may vary greatly in size, and be pedunculated or stalked-sessile, pale pink and hard upon palpation, containing fibrocytes. Fibromas consist of epithelium that is soft and circular or polyp-shaped, and the volume can often reach a very large size. When the term fibroma is used without a modifier, it is usually considered to be benign [8,9]. These type of lesions are rare and difficult to diagnose. Our case had a newly diagnosed lesion in the sinonasal tract. In the Pubmed database, we did not encounter any large-scale study focused on this type of tumors except for some case reports of fibromyxoma. For example, in the study conducted by Yalçınkaya et al. [10], 43 patients diagnosed by the Pathology Department of the Medical Faculty in Ege University and 12 patients diagnosed by the Pathology Department of the Medical Faculty in Uludag University, were investigated in the same group. As the pathological or clinical examination did not provide a definitive diagnosis in all cases, they reported that regarding the definitive diagnosis of the fibro-osseous lesions in the cranial and facial bones, the clinicopathological relationship and the radiological imaging should be considered along with the pathological parameters. This conclusion was consistent with other reports in the literature.

Regarding the findings in the literature, the concomitant consideration of the clinical and pathological findings and radiological imaging along with the pathological assessment may facilitate the diagnosis. Furthermore, a good cooperation with the patient and his/her family and a reliable history of patient are also critical. Hereby, in our case, the main objective was to draw attention to the possibility of the emergence or activation of additional pathological events besides the fracture, which may occur or become active due to the trauma. The distinctive feature of our case was not the mass itself but the immediate admission of the patient to a health center after the trauma. The cause of the swelling – edema due to the trauma or the mass itself – was another confusing finding during the examination. Therefore, in similar cases of maxillofacial trauma with persistent edema, CT and MRI examination along with the direct x-ray examination may support the diagnosis of the additional pathological events.

As far as know we couldn’t any clinical studies on both fibroma and trauma-related fibroma of the maxillar sinus in pediatric age as was detected in the literature. Wenig et al. [11] published a case with a cemento-ossifying fibroma following the maxillonasal trauma. In addition, Panta Prashanth [12] reported on fibroma-related mass development following the buccal bite or trauma in a 29-year-old patient. However, in our case – unlike to this patient – the lesion did not develop as an chronic reaction but followed a acute irritation and trauma. Nevertheless, there are more studies on ossifying fibroma in the maxillary sinus compared to the pure fibroma. The ossifying fibroma is characterized by dental calculus, plaque, microorganisms, and focal enlargement of the periodontal ligament in response to the dental apparatus or interventions. Irritation and trauma are also the main factors in these cases. The triggering mechanism for fibroma derivation from aberrant periodontal membrane or maxillary periosteum growth or development from endosteal fibrous tissue remains controversial [13] Moreover, Wenig et al. emphasized the role of the trauma in the pathophysiology of the ossifying fibroma. Thus, trauma may play a role in the development of fibroma in the pediatric age group. So fibroma, may be speculated that the trauma sustained was the critical triggering factor allowing for unchecked growth and destruction associated with an otherwise non-aggressive tumor, which may have been present prior to the traumatic incident like our case. There will be need more detailed information on this subject with meta-analysis or clinical studies involving a large number of patients. Also selection of surgical resection method depends on tumor location and size. We were performed external and endoscopic surgery as combined approached.

In conclusion, the possibility of trauma should be questioned during the history of patient in pediatric patients, who applied with a maxillofacial swelling, persistent edema. A differential diagnosis procedure for the soft tissue tumors like fibroma should be considered in the maxillofacial swelling.

## Conflicts of interest

There is not any conflict.

## Sources of funding

There is not any sponsors or any knowledgement.

## Ethical approval

This is case report which doesn’t requıre ethical approval in our institution.

## Consent

I have took from parental or guardian consent on behalf of the patient.

## Author contribution

I ‘m single author for this study that I have contrıbution of each paper that design, data collectıon, data analysis, writing paper and others.

Mehmet Akdağ, Assoc. of Professor

## Registration of research studies

A rare case report: fibroma in maxillary sinus following nasal trauma.

## Guarantor

Mehmet Akdağ.

## Provenance and peer review

Not commissioned, externally peer-reviewed.
